# Lessons for the clinical nephrologist: ureteric obstruction secondary to blood clot after kidney biopsy

**DOI:** 10.1007/s40620-021-01012-2

**Published:** 2021-04-15

**Authors:** Daniel V. O’Hara, Jeffrey K. Wong, Bruce Cooper, Germaine Wong, Muh Geot Wong, Hicham Ibrahim Cheikh Hassan

**Affiliations:** 1 Renal Unit, Wollongong Hospital, Wollongong, NSW Australia; 2 The George Institute for Global Health, UNSW, 1 King St Newtown, SydneyAustralia, NSW 2042 Australia; 3 Renal Unit, Royal North Shore Hospital, Sydney University of Sydney, Sydney, Australia; 4 Renal Unit, Liverpool Hospital, Sydney, NSW Australia; 5 Centre for Transplant and Renal Research, Westmead Hospital, Westmead, NSW Australia; 6 Centre for Kidney Research, Kids Research Institute, The Children’s Hospital, Westmead, Australia; 7 Sydney School of Public Health, University of Sydney, Sydney, NSW Australia; 8 University of Wollongong, Wollongong, NSW Australia

**Keywords:** Kidney biopsy, Renal biopsy, Ureteric Obstruction, Hemorrhage, Complication, Interventional nephrology

Kidney biopsies are a well-established, necessary tool and the gold-standard for the diagnosis and targeted management of many kidney conditions, however there are small risks of significant adverse events [1]. Potential hemorrhagic complications are prominent among these, including perinephric hematoma (11.6%), macroscopic hematuria (3.5%), bladder outlet obstruction from blood clots (0.3%), and a risk of nephrectomy (0.01%) [1]. A rare complication following a kidney biopsy is ureteric obstruction secondary to blood clots, which necessitates intervention such as ureteric stent insertion or percutaneous nephrostomy to prevent an obstructive kidney injury. Cases may be under-reported in the literature, with our systematic review of the literature identifying only 12 previously published cases of post-biopsy ureteric obstruction due to blood clot [2–11]. We report 5 new cases, describing the clinical characteristics, presentation, management and consequences, and report the findings of our systematic review of the literature, to raise awareness of this serious condition and to highlight several important learning points.

The following 5 cases involving percutaneous kidney biopsies with spring-loaded needles occurred across 4 Australian hospitals between 2015 and 2019. Pre-procedure blood pressures were < 160 mmHg systolic and < 90 mmHg diastolic. The platelet count and coagulation profiles were normal and any anticoagulants or antiplatelet medications were withheld a week prior. Further case details are summarized in Table 1.

**Table 1:** Summary of key case series details

	Case 1 ~ 60-yo Native biopsy	Case 2 ~ 70-yo Native biopsy	Case 3 ~ 60-yo Native biopsy	Case 4 ~ 60-yo Native biopsy	Case 5 ~ 60-yo Transplant biopsy
Indication for biopsy	Progressive CKD, subnephrotic proteinuria and microscopic hematuria	Progressive CKD	Progressive CKD, atrophic left kidney, proteinuria of 298 mg/mmol (2637 mg/g)	Re-staging of GPA with acute on chronic kidney failure, worsening proteinuria and active urinary sediments	Progressive kidney graft dysfunction
Imaging at biopsy	Ultrasound	Ultrasound	CT	Ultrasound	Ultrasound
Kidney side and size	Right (lower pole cyst in left kidney) 11.2 cm	Left 10.9 cm	Left 12.8 cm	Left 10 cm	Transplant 13.1 cm
Biopsy needle and number of passes	16G spring-loaded, 2 passes	16G spring-loaded, 2 passes	18G spring-loaded, 1 pass	16G spring-loaded, 4 passes	16G spring-loaded, 3 passes
Level of experience of operator	Renal advanced trainee; ~ 60 previous kidney biopsies	Renal advanced trainee; ~ 50 previous kidney biopsies	Interventional radiology fellow	Renal advanced trainee; ~ 30 previous kidney biopsies	Renal advanced trainee; 40 previous kidney biopsies, supervised by a highly experienced nephrologist
Antiplatelets or anticoagulants	Nil	Nil	Nil	Nil	Aspirin withheld 1 week prior
Urea pre-biopsy (mmol/L)	9.2 (25.8 mg/dL)	26.5 (74.2 mg/dL)	37.7 (105.6 mg/dL)	25.0 (70.0 mg/dL)	22.8 (63.9 mg/dL)
eGFR pre-biopsy (mL/min/1.73m^2^)	68	10	9	25	21
Desmopressin pre-biopsy	No	No	No	No	Yes
Presence of immediate post-biopsy hematuria	Yes	Yes	Yes	Yes	Yes
Suspected timing of obstruction	5 days after biopsy	9 days after biopsy	< 24 h	< 24 h	< 24 h
Intervention required	Ureteric stent, removed 6 weeks later	Ureteric stent, removed 4 weeks later	Ureteric stent, removed 2 months later	Ureteric stent, removed 3 months later	Indwelling catheter inserted with continuous bladder irrigation, proceeded to nephrostomy which was removed 3 days later
Biopsy result	Non-specific mesangio-pathic changes	Diabetic and hypertensive nephropathy	IgA nephropathy	Focal necrotising glomerulo-nephritis	Moderate glomerulitis, C4D negative, moderate interstitial and tubular fibrosis
Presence of vessels in biopsy	No	No	Yes	Yes	Yes
Additional notes	A coagulopathy and thrombo-philia panel were normal				

## Case 1

**Fig. 1:**
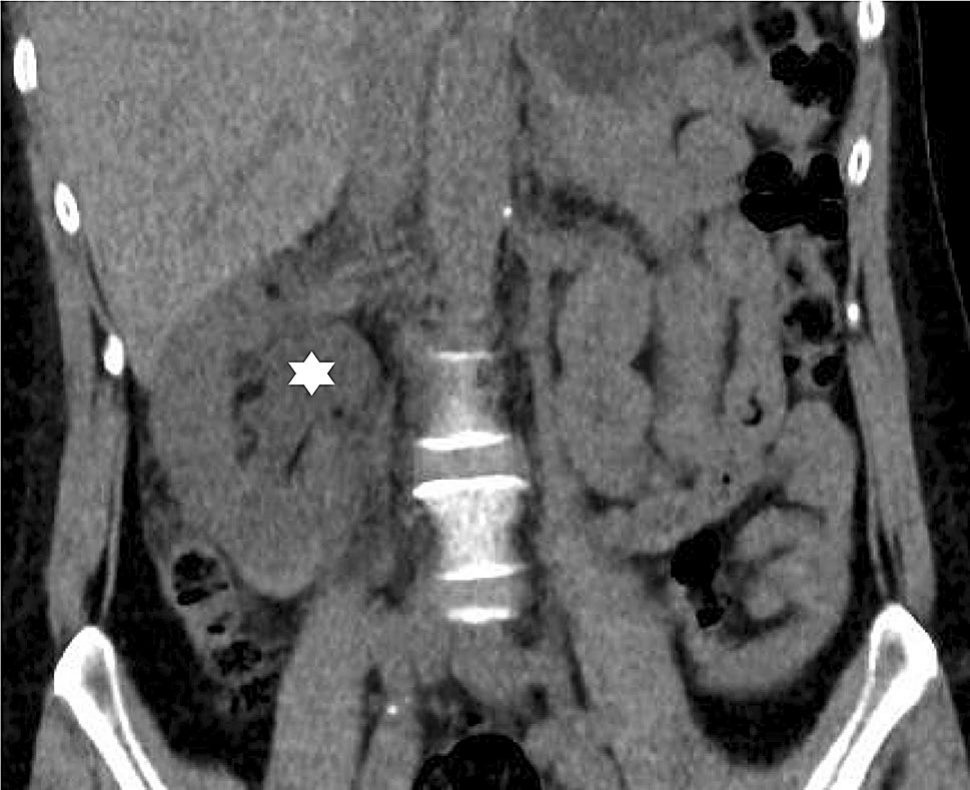
Non-contrast CT of the patient’s abdomen demonstrating right-sided hydronephrosis (asterisk)

A ~ 60-year-old patient underwent a right native kidney biopsy for investigation of worsening chronic kidney disease (CKD) with an estimated glomerular filtration rate (eGFR) of 68 mL/min/1.73m^2^ down from 82 mL/min/1.73m^2^ a year prior, proteinuria of 61 mg/mmol (539.8 mg/g) and microscopic hematuria, on a background of untreated mild systemic lupus erythematosus. Macroscopic hematuria developed immediately post-biopsy and resolved within 6 h with intravenous hydration. After discharge with appropriate safety instructions, the patient developed a 1 h self-resolving episode of right flank pain overnight. Severe right flank pain recurred on day 5 post-biopsy, together with macroscopic frank hematuria. A CT scan (Fig. 1) confirmed hydronephrosis and a ureteric blood clot. Symptoms resolved following ureteric stent insertion.

### Case 2

A ~ 70-year-old patient underwent a left native kidney biopsy for rapidly progressing CKD with eGFR 10 mL/min/1.73m^2^. The patient was hospitalized and managed conservatively for immediate post-biopsy frank hematuria that continued for 2 days. Hematuria recurred 9 days post-biopsy, accompanied by flank pain. A three-way urinary catheter and irrigation were required for bladder clot retention. A CT scan showed blood products in the left kidney pelvis and proximal ureter. Conservative management was attempted, however early signs of sepsis soon prompted ureteric stent insertion.

### Case 3

A ~ 60-year-old patient underwent a native right kidney biopsy for quickly progressing CKD with eGFR 9 mL/min/1.73m^2^, performed under CT guidance due to obesity (BMI 48.1 kg/m^2^). Hematuria and hypotension developed an hour post-biopsy. A CT scan demonstrated fresh blood in the right ureter and bladder with right-sided hydronephrosis. A ureteric stent was inserted for acute on chronic kidney failure, with immediate improvement in kidney function.

### Case 4

A ~ 60-year-old patient presented for investigation of acute kidney injury, epistaxis and high proteinase 3 (PR3) antibody, on a background of known granulomatosis with polyangiitis (GPA). Immediate post-biopsy macroscopic hematuria continued for 3 days in association with deteriorating kidney function. A CT showed ipsilateral hydronephrosis and hydroureter. At cystoscopy a blood clot cast was found protruding from the ipsilateral ureteric orifice, which was extracted with biopsy forceps. A ureteric stent was inserted with subsequent improvement in kidney function.

### Case 5

A ~ 60-year-old kidney transplant recipient had a transplant kidney biopsy for graft dysfunction with suspected antibody-mediated rejection. The eGFR was 21 mL/min/1.73m^2^ with urea 22.8 mmol/L (63.9 mg/dL) and body weight of 116 kg. Desmopressin (Deamino-8-d-arginine vasopressin; DDAVP) 20 µg was administered intravenously just prior to the biopsy. Immediate post-biopsy hematuria resulted in urinary retention. A further 20 µg desmopressin was given. An ultrasound demonstrated transplant hydronephrosis with a large bladder clot requiring continuous bladder irrigation and regular bladder washouts. Hydronephrosis persisted for 3 days, with worsening kidney function. A percutaneous nephrostomy was performed, as there was concern that retrograde stent insertion would be technically difficult for the transplanted ureter. Three days later a contrast antegrade nephrostogram demonstrated free drainage of contrast into the bladder without need for a ureteric stent, and the nephrostomy tube was removed. Several days after these events, the patient had sudden cardiac arrest with pulseless electrical activity and died. The patient’s usual aspirin, taken to preserve the patency of a transplant kidney artery stent, had been withheld since 1 week prior to the biopsy.

## Lessons for the clinical nephrologist

Ureteric obstruction is a rare complication of kidney biopsies. We conducted a systematic review of the literature, using multiple combinations of Medline and Embase search terms including kidney biopsy, renal biopsy, complication, hematuria, hydronephrosis, ureteric obstruction, ureteral obstruction, obstruction, and safety, and only identified 12 published cases over a 35 year time period (Table S1, Supplementary Appendix) [2–11]. This complication may be under-reported however, as is shown by the fact that we have identified 5 occurrences in our hospitals within recent years. In light of the significance of this complication, and guided by our experience and the information provided by the published cases, we present the following learning points.

### Immediate hematuria is usually observed

All of our cases of ureteric obstruction involved frank hematuria immediately post- biopsy, and this was also seen in at least 7 of the 12 published cases (Table S1). Post-biopsy frank hematuria should therefore raise clinical suspicion of this potential complication.

### Ureteric obstruction may be a delayed complication or a delayed diagnosis

The timing of presentations ranged from < 24 h in published cases up to 9 days post-biopsy in our Case 1, and 25 days post-biopsy in a case reported by Bergman and colleagues [2]. The delay is atypical of other hemorrhagic complications of kidney biopsy, with up to 89% of all biopsy complications presenting within the first 24 h [12]. The delay in presentation of hydronephrosis could relate to an intracalyceal thrombus which becomes dislodged at a later time, thereby causing obstruction. The time it takes to observe a creatinine increase may also delay the diagnosis. Such delays can have significant implications for patient care, particularly if patients return to geographically isolated areas soon after their biopsy. We recommend imaging for patients who re-develop frank hematuria or flank pain to exclude hydronephrosis.

### Intervention is required

All patients in our case series required additional intervention, as did all the cases described in the literature where the patient outcome was discussed (Table S1).

For the cases involving native kidney biopsies, ureteric stents were required in at least 3 of the 4 published incidences of the complication; the patient outcome was not discussed in the remaining case [5]. In 3 cases, retrograde ureteric instillation of thrombolytics was required. Bergman and colleagues report using the ureteric catheter to instill streptokinase twice daily for a total of 5 treatments, after the stent had stopped draining [2]. By 4 days after intervention the pain had fully resolved and there was no further clot seen on imaging. Stegmayr and colleagues also describe using retrograde ureteric instillation of streptokinase [3]. Grabe and colleagues describe using crystalline trypsin for a similar case, after 2 unsuccessful attempts to relieve hydronephrosis with ureteric stents [4].

For the cases involving transplant kidney biopsies, percutaneous nephrostomy tubes are usually required. Tsai and colleagues report the successful use of a nephrostomy tube with removal one week later [6]. Four other published cases describe the successful use of nephrostomy tubes without detailing the timing of removal [9, 10]. Chan and colleagues discuss a patient who was taken for insertion of a percutaneous nephrostomy tube, but during the percutaneous injection of contrast for this procedure the clot was dislodged and the obstruction was relieved [8]. Boschiero and colleagues report resolution after insertion of a transplant ureteric stent, [7], while McDonald and colleagues did not describe their patient’s management [11].

### Advanced kidney disease is a risk factor

It is well known that advanced kidney disease increases the risk of hemorrhagic complications of kidney biopsies [1]. In 4 of our 5 cases, the eGFR was 25 mL/min/1.73m^2^ or less and urea was > 22 mmol/L (> 61 mg/dL). Clinicians were aware of this higher risk and felt that the histological information would guide clinical management, including whether commencement or escalation of immunosuppression was required. All kidneys were at least 10 cm in size.

Only one patient (Case 5) was given desmopressin to reduce uremic bleeding risk. The evidence for desmopressin in reducing uremic bleeding remains extremely limited. It has been seen to temporarily reduce in vitro bleeding time in patients on hemodialysis or with creatinine > 619 μmol/L (> 7 mg/dL) [13], but 3 single-center case series have each produced no convincing evidence of reduced rates of hemorrhage after kidney biopsy [14–16], and there is an approximately 7% risk of severe hyponatremia [17]. Current guidelines give no recommendation for desmopressin on the grounds of inadequate evidence [18–20].

### Other risk factors

There were no clear trends in patient gender, the number of biopsy passes, or the kidney pathology on biopsy. Three of the 5 biopsies included vessels in the biopsy sample.

There is conflicting evidence regarding whether the risk of bleeding complications is increased when biopsies are performed by trainees. While one retrospective study of 159 inpatient biopsies identified a higher risk of blood transfusion requirement with biopsies performed by trainees [21], two other studies have demonstrated similar complication rates and diagnostic accuracy compared to biopsies performed by nephrologists and/or interventional radiologists [22, 23]. In our experience it is common practice in Australia for kidney biopsies to be performed by trainees, and all proceduralists had performed at least 30 biopsies previously.

### Other complications may subsequently occur

In 4 of our 5 cases, the ureteric obstruction resulted in an acute kidney injury. In our Case 5, the bleeding complications necessitated a delay in returning to aspirin use, which may have contributed to a fatal acute coronary event, although the aspirin had been taken to preserve the patency of a transplant kidney artery stent, rather than for a cardiovascular indication. In Case 5, there was concern for sepsis when the patient became febrile, however this resolved with stent insertion and an antibiotic course. Stegmayr and colleagues describe a patient who developed urosepsis and an acute myocardial infarction during an admission for ureteric obstruction [3].

### Ureteric obstruction should be discussed in the consent process

Current guidelines discuss the risk of hematuria, perinephric hematoma formation and the risk of requiring blood transfusion, but not the risk of acute ureteric obstruction from blood clot [18–20]. As this complication is a devastating event which necessitates procedural intervention, it should be listed as a potential complication in kidney biopsy guidelines to raise awareness among proceduralists and the nephrology community, and it should be discussed and included in the informed consent process.

In summary, ureteric obstruction secondary to hematuria after kidney biopsy is a rare but serious complication that is under-reported in the literature. It warrants consideration when consenting and advising patients about kidney biopsies, and when planning and managing the procedure and post-biopsy care, and should be considered in future medical research exploring kidney biopsy safety.

## Supplementary Information

Below is the link to the electronic supplementary material.

Supplementary file1 (DOCX 30 KB)
