# Supplementary material for: Lessons for the clinical nephrologist: ureteric obstruction secondary to blood clot after kidney biopsy
Source: J Nephrol. 2021 Apr 15;34(6):2131–6. doi: 10.1007/s40620-021-01012-2 (PMC8610936; doi:10.1007/s40620-021-01012-2)
Supplement: Supplementary file 1 — Supplementary file1 (DOCX 30 KB) [file 40620_2021_1012_MOESM1_ESM.docx]

**Supplementary Appendix**

**Table S1:** Published cases of ureteric obstruction after kidney biopsy

| **Author**  **and Year** | **Demographics** | **Indication** | **Type of Biopsy** | **Bleeding Risks** | **Intervention Required** | **Days Between Biopsy and Diagnosis** | **Immediate Hematuria** | **Biopsy Result** |
| --- | --- | --- | --- | --- | --- | --- | --- | --- |
| Bergman 1990  (2) | 41yo male | Nephrotic syndrome | Percutaneous right native kidney then open left kidney biopsy | Blood urea nitrogen 3.7mg/dL (normal 0.8-1.4) | Right-sided ureteric stent with streptokinase instillation | 25 days | Yes | Nil glomeruli on the percutaneous biopsy. Subsequent open left kidney biopsy showed minimal change disease |
| Stegmayr 1984  (3) | 47yo male | Nephrotic syndrome | Percutaneous right native kidney biopsy | Nil stated | Ureteric stent with instillation of a serum and streptokinase solution. IV antibiotics were administered | 5 days | Yes | Mesangiocapillary glomerulonephritis |
| Grabe  1986  (4) | 22yo male | Hypertension and rapid deterioration of kidney function on background of previous mesangio-proliferative glomerulo-nephritis | Percutaneous right native kidney biopsy | Nil stated | 2 attempts at ureteric stent insertion then ureteric instillation of trypsin. Stent removed 4 days later. Brief requirement for hemodialysis | 24 hours | Yes | Not discussed |
| Birnholz 1985  (5) | Not discussed | Not discussed | Percutaneous ultrasound-guided native kidney biopsy with a non-spring-loaded needle | Not discussed | Not discussed | Not discussed | Not discussed | Not discussed |
| Tsai  2016  (6) | Female, age not discussed | Not discussed | Ultrasound-guided percutaneous transplant kidney biopsy with spring-loaded 16 or 18 gauge needle | Not discussed | Percutaneous nephrostomy | 1 day | Yes | Not discussed |
| Boschiero 1985  (7) | Not discussed | Not discussed | Percutaneous transplant kidney biopsy with a non-spring-loaded needle, unclear if ultrasound guidance used | Not discussed | Ureteric stent | 7 days | No | Not discussed |
| Chan  2000  (8) | Female, age not discussed | Not discussed | Ultrasound-guided percutaneous transplant kidney with 16-gauge spring-loaded needle | Not discussed | Referred for percutaneous nephrostomy catheter insertion but obstruction relieved during initial nephrosto-gram | Not discussed | Yes | Not discussed |
| Wilczek 1990  (9) | 3 cases, demo-graphics not discussed | Not discussed | Percutaneous transplant kidney with non-spring loaded needle and without ultrasound guidance | Not discussed | Not discussed | Not discussed | Not discussed | Not discussed |
| Schmid 2013  (10) | Not discussed | Unexplained reduced graft function | Transvenous transplant kidney biopsy | Taking aspirin | Percutaneous nephrostomy; note known transplant ureteric stenosis | 2 days | Yes | Not discussed |
| McDonald 1993  (11) | Not discussed | Not discussed | Ultrasound-guided, percutaneous transplant kidney biopsy with spring-loaded needle | Authors report that needle was inserted too far into kidney parenchyma before firing | Ureteric stent and blood transfusion | Not discussed | Yes | Not discussed |

**References**

1. Corapi KM, Chen JLT, Balk EM, Gordon CE. Bleeding complications of native kidney biopsy: A systematic review and meta-analysis. Am J Kidney Dis. 2012;60(1):62-73.

2. Bergman SM, Frentz GD, Wallin JD. Ureteral obstruction due to blood clot following percutaneous renal biopsy: resolution with intraureteral streptokinase. J Urol. 1990;143(1):113-5.

3. Stegmayr B, Orsten PA. Lysis of obstructive renal pelvic clots with retrograde instillation of streptokinase: A case report. Scand J Urol Nephrol. 1984;18(4):347-50.

4. Grabe M, Forsberg B. Retrograde trypsin instillation into the renal pelvis for the dissolution of obstructive blood clots. Eur Urol. 1986;12(1):69-70.

5. Birnholz JC, Kasinath BS, Corwin HL. An improved technique for ultrasound guided percutaneous renal biopsy. Kidney Int. 1985;27(1):80-2.

6. Tsai SF, Chen CH, Shu KH, Cheng CH, Yu TM, Chuang YW, et al. Current safety of renal allograft biopsy with indication in adult recipients: an observational study. Medicine. 2016;95(6):e2816.

7. Boschiero LB, Saggin P, Galante O, Prati GF, Dean P, Longo M, et al. Renal needle biopsy of the transplant kidney: vascular and urologic complications. Urol Int. 1992;48(2):130-3.

8. Chan R, Common AA, Marcuzzi D. Ultrasound-guided renal biopsy: experience using an automated core biopsy system. Can Assoc Radiol J. 2000;51(2):107-13.

9. Wilczek HE. Percutaneous needle biopsy of the renal allograft. A clinical safety evaluation of 1129 biopsies. Transplantation. 1990;50(5):790-7.

10. Schmid A, Jacobi J, Kuefner MA, Lell M, Wuest W, Mayer-Kadner I, et al. Transvenous renal transplant biopsy via a transfemoral approach. Am J Transplant. 2013;13(5):1262-71.

11. McDonald MW, Sosnowski JT, Mahin EJ, Willard DA, Lamm DL. Automatic spring-loaded biopsy gun with ultrasonic control for renal transplant biopsy. Urology. 1993;42(5):580-2.

12. Whittier WL, Korbet SM. Timing of complications in percutaneous renal biopsy. J Am Soc Nephrol. 2004;15(1):142-7.

13. Mannucci PMMD, Remuzzi GMD, Pusineri FMD, Lombardi RB, Valsecchi CB, Mecca GMD, et al. Deamino-8-D-arginine vasopressin shortens the bleeding time in uremia. N Engl J Med. 1983;308(1):8-12.

14. Peters B, Hadimeri H, Molne J, Nasic S, Jensen G, Stegmayr B. Desmopressin (Octostim) before a native kidney biopsy can reduce the risk for biopsy complications in patients with impaired renal function: A pilot study. Nephrology. 2018;23(4):366-70.

15. Ho QY, Lim CC, Thangaraju S, Siow B, Chin YM, Hao Y, et al. Bleeding complications and adverse events after desmopressin acetate for percutaneous renal transplant biopsy. Ann Acad Med Singapore. 2020;49(2):52-64.

16. Athavale A, Kulkarni H, Arslan CD, Hart P. Desmopressin and bleeding risk after percutaneous kidney biopsy. BMC Nephrol. 2019;20 (1) (no pagination)(413).

17. Lim CC, Siow B, Choo JCJ, Chawla M, Chin YM, Kee T, et al. Desmopressin for the prevention of bleeding in percutaneous kidney biopsy: efficacy and hyponatremia. Int Urol Nephrol. 2019;51(6):995-1004.

18. Hogan JJ, Mocanu M, Berns JS. The native kidney biopsy: update and evidence for best practice. Clin J Am Soc Nephrol. 2016;11(2):354-62.

19. Luciano RL, Moeckel GW. Update on the native kidney biopsy: core curriculum 2019. Am J Kidney Dis. 2019;73(3):404-15.

20. MacGinley R, Champion De Crespigny PJ, Gutman T, Lopez-Vargas P, Manera K, Menahem S, et al. KHA-CARI guideline recommendations for renal biopsy. Nephrology (Carlton, Vic). 2019;24(12):1205-13.

21. Moledina DG, Luciano RL, Kukova L, Chan L, Saha A, Nadkarni G, et al. Kidney biopsy–related complications in hospitalized patients with acute kidney disease. Clin J Am Soc Nephrol. 2018;13(11):1633-40.

22. Bapat M, Martin TC, Kattamanchi S, Tokita JE, Sharma S, Nadkarni GN, et al. Safety of kidney biopsy performed by nephrology trainees vs interventional radiology. J Am Soc Nephrol. 2018;29:785.

23. Basu S, Hamour S, Moochhala S. Renal biopsy complication rates and transfusion requirements in a tertiary teaching hospital. Nephrol Dial Transplant. 2018;33 (Supplement 1):i25.
